# Graphene-Enhanced Fluoroelastomer Composites for Advanced Applications

**DOI:** 10.1021/acsomega.5c08944

**Published:** 2026-02-20

**Authors:** Ramon Mendonça Teles, Daiana Cristina Metz Arnold, Marco Antônio Siqueira Rodrigues, Diana Exenberger Finkler, Carlos Leonardo Pandolfo Carone

**Affiliations:** † Graduate Program in Materials Technology and Industrial Processes, 125098 Feevale University, Av. Edgar Hoffmeister, No. 600, Pavilion No. 03, Room No. 02, Valetec, Campo Bom, RS 93700-000, Brazil; ‡ Serall Ltda. Av. Edgar Hoffmeister, No. 600−Pavilion No. 03, Room No. 02−Valetec, Campo Bom, RS 93700-000, Brazil

## Abstract

Fluoroelastomers are widely used in applications requiring resistance to high temperatures, aggressive chemicals, and elevated pressure conditions, enabling efficient applications in harsh environments. The incorporation of graphene has shown potential to enhance the mechanical and thermal performance, resulting in more efficient composites. However, graphene incorporation remains a challenge due to the difficulty of dispersing graphene sheets within the rubber matrix. This research developed fluoroelastomer composites with 1, 2, and 3 phr of graphene using both the melt blending method and the solvent-assisted method with acetonitrile to incorporate graphene. The composites were characterized by Fourier transform infrared spectroscopy (FT-IR), scanning electron microscopy (SEM), and energy-dispersive X-ray spectrometry (EDS), thermogravimetric analysis (TGA), and dynamic mechanical analysis (DMA), as well as Shore A hardness and tensile testing. FT-IR indicated the complete removal of acetonitrile and similar spectra among all of the composites, indicating that the solvent method does not chemically modify the samples. SEM and EDS analyses revealed overall similar morphologies among the samples; however, composites containing 2 and 3 phr of graphene processed via the solvent-assisted method exhibited a more pronounced surface roughness. TGA indicated up to a 38% increase in initial degradation resistance in composites with 2 and 3 phr incorporated via the solvent method. In dynamic mechanical analysis (DMA) tests, samples with 3 phr exhibited higher energy dissipation at −30 °C and a higher *T*_g_ (11.8 °C) when prepared using the solvent method. Shore A hardness decreased by up to 11.8% in samples from the standard method. In tensile testing, the 3 phr sample via solvent incorporation exhibited the best performance, with a tensile strength of 21.74 MPa and intermediate elongation. These results indicate that the improvements achieved through enhanced graphene dispersion result from restricted molecular chain mobility and more efficient stress transfer, enabled by strengthened interactions between graphene and the polymer matrix. Overall, these findings emphasize the importance of developing more robust and efficient rubber composites to address the growing performance requirements of modern material applications.

## Graphical abstract

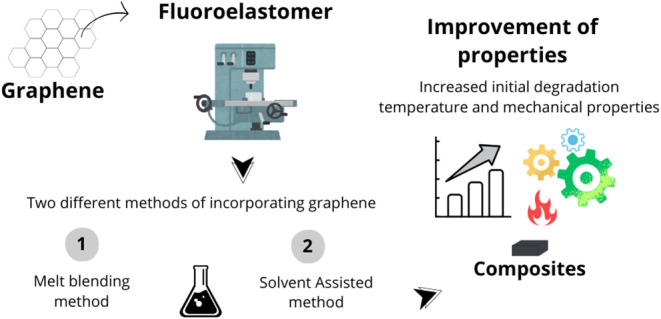

## 1 Introduction

The search for new high-performance rubber formulations has intensified, driven by the growing demand for more specific and challenging applications. Technological innovation aims to overcome the limitations of existing materials, fostering the development of new composites that offer greater efficiency, comfort, and safety in a wide range of industrial and technological applications.^1–5^ Among these new materials, fluoroelastomer-based composites have stood out for combining different properties, providing improvements in both mechanical and thermal performance.^6–8^

Fluoroelastomers, developed in the 1950s, emerged as a response to the demands of harsh environments, where oils, gases, and high temperatures are present. These elastomers exhibit remarkable chemical and mechanical resistance and are widely used in sectors requiring durability under extreme conditions. However, the search for new high-performance rubber materials continues, aiming to enhance mechanical resistance and performance in demanding environments.^9,10^

The incorporation of graphene into fluoroelastomers represents an innovative approach to develop composites with enhanced properties. Understanding how graphene particles interact with the elastomeric matrix enables optimization of the nanofiller utilization, aiming to avoid agglomerates in the composite, known as clusters. Since the properties of this new material depend on this interaction, ensuring good dispersion can lead to improved results.^11,12^ To achieve such properties, several fluoroelastomer formulations have been investigated,^7,10,13–17^ including the addition of specific fillers that improve composite performance. Among these fillers, carbon-based nanomaterials have gained increasing attention due to their ability to enhance mechanical, thermal, and functional properties even at low loadings. In this context, graphene stands out as a promising reinforcement because of its exceptional intrinsic properties and strong potential for interaction with rubber matrices, which will be discussed in the following paragraph.^8,18,19^

Graphene has gained attention as a potential additive, due to its large surface area and good crystalline structure with few defects, making them suitable for high-performance applications.^20,21^ A substantial number of studies have concentrated on the incorporation of graphene oxide, primarily due to its relatively easier dispersion within rubber matrices and the presence of oxygen-containing functional groups that enhance interfacial interactions.^8,15,22^ In contrast, investigations involving graphene are still limited, particularly in fluoroelastomer systems, where issues related to dispersion, interfacial compatibility, and processing complexity hinder broader applications. As a result, the incorporation of graphene into fluoroelastomers remains an underexplored research area, despite its significant potential to achieve enhanced and multifunctional properties without making major modifications to the graphene.^7,13,14^

Graphene is the monolayer of carbon atoms arranged in a two-dimensional (2D) structure, with mechanical strength 200 times greater than steel, it has attracted attention due to its unique properties such as high thermal, electrical conductivity and low density.^23,24^ These characteristics enable diverse applications, particularly in the development of stronger and more durable materials.^25–27^ Currently regarded as the thinnest material in the world, it presents a planar structure with the thickness of only one carbon atom. Essentially, it can be visualized as a single layer of graphite since the stacking of graphene particles driven by π–π interactions forms graphite, which tends to bring particles closer together through these interactions. Its composition is characterized by sp^2^–sp^2^ covalent bonds, which confer excellent electrical conductivity and remarkable mechanical strength.^26^

The dispersion of graphene within the rubber matrix is a critical factor. When it is poorly dispersed, it tends to form agglomerates due to its high surface area and interactions between the graphene sheets, specifically, π–π interactions. These agglomerates act as defects within the rubber matrix, potentially reducing mechanical strength, increasing brittleness, and compromising the homogeneity of the composite’s final properties.^12,27^ In composites with poor dispersion, load and stress transfer between the matrix and the reinforcement is inefficient.^28^

The development of fluoroelastomer composites incorporating graphene can be an efficient solution to enhance the performance of materials in advanced applications. The incorporation of graphene and its derivatives, such as graphene oxide and modified graphene, enables the production of composites with improved mechanical and thermal properties, ideal for demanding operational environments, such as those found in the oil industry, for example. These additives contribute to improvements in tensile strength, hardness, and thermal stability while also reducing gas permeability and enhancing chemical and wear resistance. To maximize these results, compatibilization with the matrix and effective dispersion of graphene are essential factors.^8,13,15^

To optimize the properties of graphene-based composites, the particles must be well dispersed, and in some applications, conductive pathways within the matrix must be established to enable properties such as electrical conductivity. Figure 1 presents a schematic representation of the fluoroelastomer-based composite with graphene incorporation, highlighting the aspects of dispersion. Even with modified graphene, if the dispersion is not efficient, there is a chance that the desired best properties for the composite will not be obtained.

**Figure 1:**
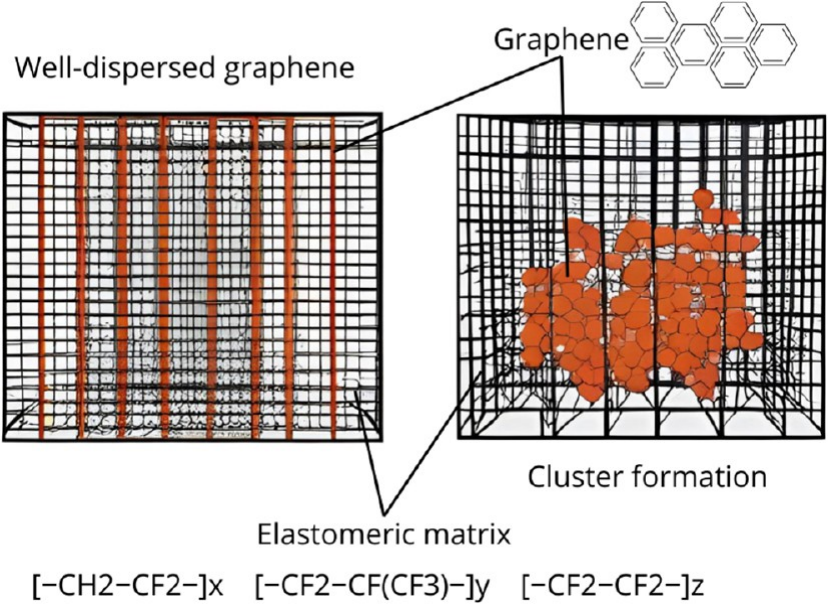
Graphene dispersion in a fluoroelastomer rubber. Source—Prepared by the authors (2025).

The scheme highlights two distinct behaviors: homogeneous dispersion, which promotes better integration between the matrix and the reinforcement, and agglomerate formation, which compromises the uniformity of the composite. Thus, the figure illustrates how the state of graphene dispersion within the matrix can decisively influence the structural and functional properties of the material, creating points of structural brittleness in the composite.

With improved graphene dispersion, the surface area available for contact with the matrix increases, expanding the interfacial adhesion regions and strengthening mechanisms such as physical anchoring and stress transfer. This more efficient interaction favors the formation of a continuous load transfer network within the fluoroelastomer, resulting in better mechanical and dynamic-mechanical properties, as reported by Zhao et al.,^29^ who demonstrated that enhanced interfacial compatibility and synergistic filler effects in FKM-based composites significantly improved tensile performance and viscoelastic behavior due to more effective stress transfer and matrix–filler interaction.

^15^Wei et al.^15^ evaluated five different solvents for the dispersion of graphene oxide in a fluoroelastomer matrix and concluded that acetonitrile is one of the most attractive solvents for this purpose, as it promotes effective dispersion and helps the interfacial interaction between the filler and the FKM matrix. In a subsequent study,^15,16^ compared graphene oxide and reduced graphene in fluoroelastomer composites, noting that graphene oxide offers a more economical route for filler production, while reduced graphene demonstrated superior mechanical properties, attributed to its higher structural integrity and more efficient stress transfer within the polymer matrix.

In this sense, acetonitrile, being a polar aprotic solvent with good affinity for the graphene surface and capable of temporarily reducing the van der Waals forces that keep the lamellae aggregated, can be an attractive solvent for dispersion. Therefore, during agitation or sonication, it facilitates the separation of the sheets and improves the dispersion of the filler before incorporation into FKM. Studies on solvent-graphene adhesion show that solvents with a higher polar component, such as acetonitrile, exhibit greater adhesion work, favoring the momentary stability of the suspension and a more homogeneous dispersion of the graphene.^30^

Consequently, researchers seek alternatives to disperse graphene in rubber matrixes in order to achieve a more homogeneous distribution, reduce agglomerate formation, and thereby optimize interfacial interactions. This improvement in dispersion aims to enhance the mechanical, thermal, and electrical properties of the composite, broadening its applications under severe operating conditions. Given these challenges and opportunities, this research aims to develop fluoroelastomer composites by incorporating graphene into the matrix. The goal is to explore the potential of this material in enhancing the mechanical, thermal, and chemical properties of the composites, making them more suitable for a wide range of advanced applications.

## 2 Materials and Methods

This section describes the materials and experimental procedures employed in this study. The compositions and formulations of the samples prepared using the two different processing methods are presented in Table 1.

**Table 1:** Formulations of the samples developed by two methods^a^

	melt blending method (phr)			solvent method (phr)			
sample	ref	FG1	FG2	FG3	FAG1	FAG2	FAG3
florelastomer	100.00	100.00	100.00	100.00	100.00	100.00	100.00
carbon Black	13.00	13.00	13.00	13.00	13.00	13.00	13.00
carnauba wax	2.50	2.50	2.50	2.50	2.50	2.50	2.50
peroxide	1.50	1.50	1.50	1.50	1.50	1.50	1.50
co-agent	10.00	10.00	10.00	10.00	10.00	10.00	10.00
graphene	0.00	1.00	2.00	3.00	1.00	2.00	3.00

a Source—Prepared by the authors (2025).

For the development of the formulations, the steps illustrated in Figure 2 were followed for processing the composites with a fluoroelastomer matrix and incorporation of 1 phr, 2 phr, and 3 phr of graphene using two distinct methods. A sample without graphene was developed for comparison (the reference sample).

**Figure 2:**
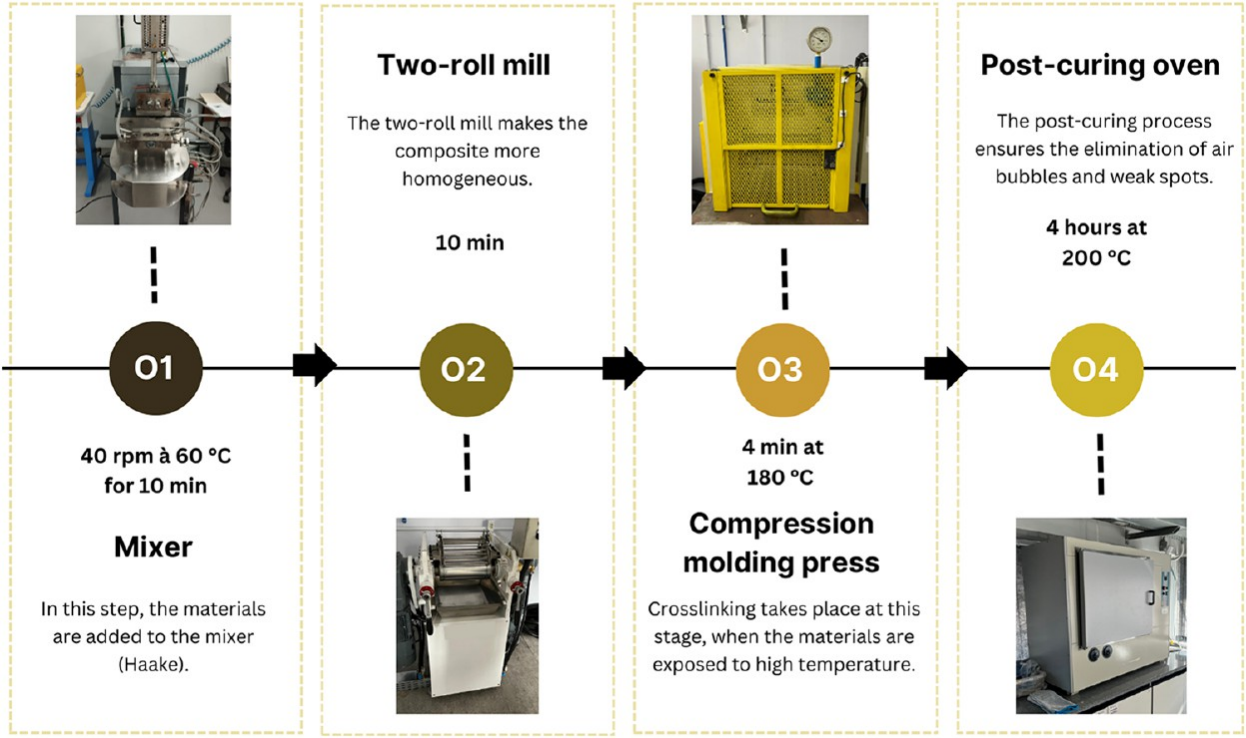
Development process. Source—Prepared by the authors (2025).

The melt blending method for incorporating graphene into elastomeric matrices is based on melt mixing in closed-type Haake mixers. This process is widely used in the preparation of rubber composites, as it allows dispersion of the fillers directly in the matrix, without the need for prior functionalization or graphene dispersion steps, but this dispersion happens in a limited way. These samples were identified as FG1, FG2, and FG3, with the numbers representing the amount of graphene in phr.

The second method was adapted from a study conducted by Wei et al.^15^ For the graphene incorporation, 200 mL of acetonitrile was initially measured and placed in a three-neck round-bottom flask together with the elastomer fragmented into small portions. This mixture was kept under continuous magnetic stirring for 72 h, at speed 4 on the speed scale of the Quimis equipment. In parallel, 100 mL of acetonitrile were mixed with the corresponding amount of graphene in phr, and the dispersion was magnetically stirred for 24 h. Finally, the elastomer with incorporated graphene was filtered and dried in an drying oven for 48 h at 50 °C. For this method, the samples were identified as FAG1, FAG2, and FAG3, with the numbers representing the amount of graphene in phr.

Graphene was characterized by Raman spectroscopy, transmission electron microscopy (TEM) using a TECNAI G2 T20 FEI microscope operating at 200 kV, and scanning electron microscopy (SEM) using a JEOL scanning electron microscope, model JSM6510LV, at 10 kV.

The developed composites were characterized by Fourier transform infrared spectroscopy (FT-IR) using a Spectrum Two infrared spectrophotometer (PerkinElmer).

Morphological analysis was carried out using a scanning electron microscope (SEM, JEOL, JSM-6010LA) equipped with secondary electron and backscattered electron detectors. Prior to analysis, the samples were coated with a thin carbon layer to ensure a surface conductivity. Energy-dispersive X-ray spectroscopy (EDS) was employed for elemental analysis and chemical characterization of the composites.

Thermal analysis was carried out by thermogravimetric analysis (TGA) using a Shimadzu TGA 51H thermogravimetric analyzer with a temperature range from 23 to 1000 °C at a heating rate of 10 °C/min under a nitrogen flow of 50 mL/min, and DMA using a Mtravib DMA 25/50 dynamic mechanical analyzer at a frequency of 2 Hz, displacement amplitude of 20 μm, in tensile mode, from −30 to 100 °C with a heating rate of 3 °C/min.

Mechanical analysis was carried out by measuring Shore A hardness using a MEDTEC durometer, and tensile strength and elongation tests were conducted using a Maqtest dynamometer with a 50 kgf load cell.

## 3 Results and Discussion

In this section, the results of the graphene characterization tests, as well as the analyses of the composites prepared with the fluoroelastomer matrix incorporating graphene are presented.

### 3.1 Graphene Characterization

Figure 3 shows the results of the Raman spectroscopy analysis, highlighting the main characteristic peaks and the structural information on the material, such as defects, stacking, and the number of layers.

**Figure 3:**
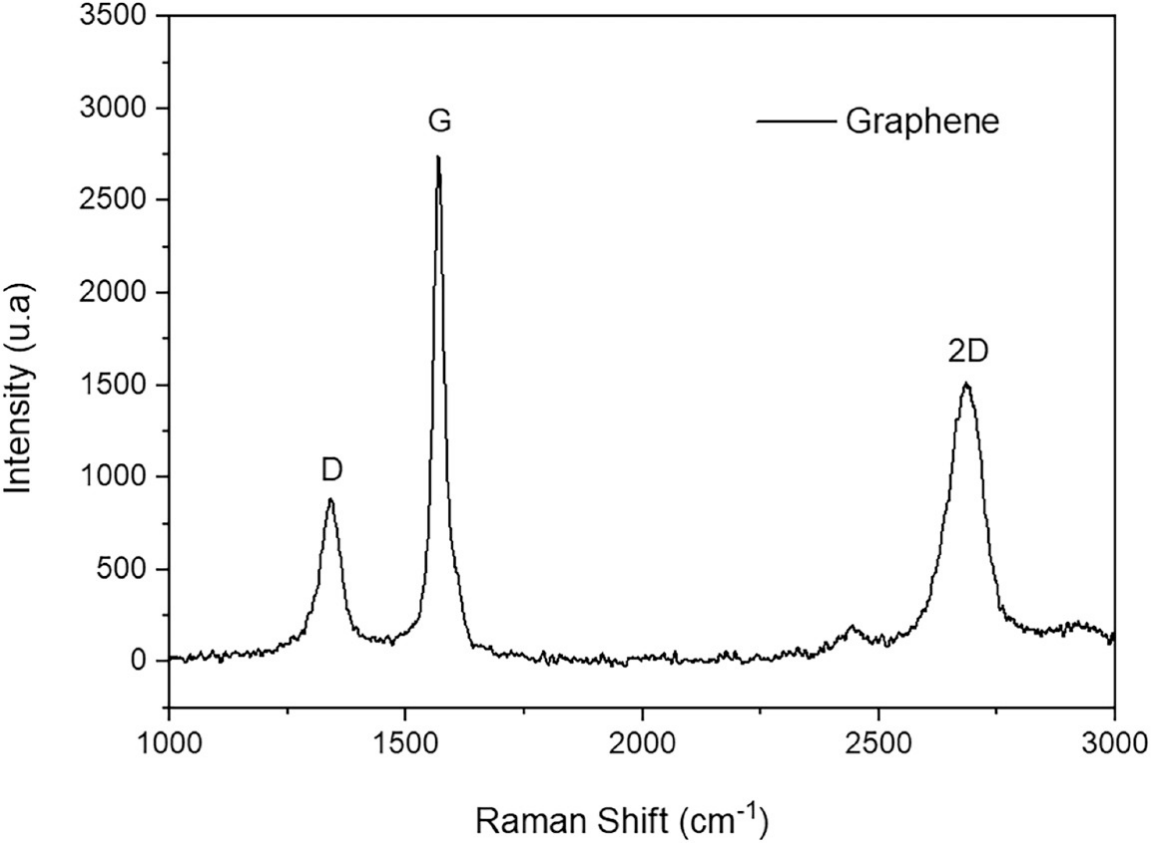
Raman spectrum of the graphene sample. Source—Prepared by the authors (2025).

This technique is used to evaluate the structure, defect density, and layer number of graphene. In the presented spectrum, three characteristic bands are observed: the D band, around 1350 cm^–1^ with 880 au, the G band, near 1580 cm^–1^ with 2743 au, and the 2D band, around 2680 cm^–1^ with 1518 au The G band is associated with the vibrations of sp^2^-hybridized carbon atoms, where each carbon is bonded to three atoms in a plane with 120° angles, while the D band indicates the presence of defects or disorder in the structure.^30^ The 2D band is the second order of the D band and provides information regarding the number of layers.^12^

The TEM analysis enabled the characterization of graphene on the nanometric scale. Figure 4 displays graphene sheets with translucent regions, folds, and overlaps, confirming their lamellar and two-dimensional nature.

**Figure 4:**
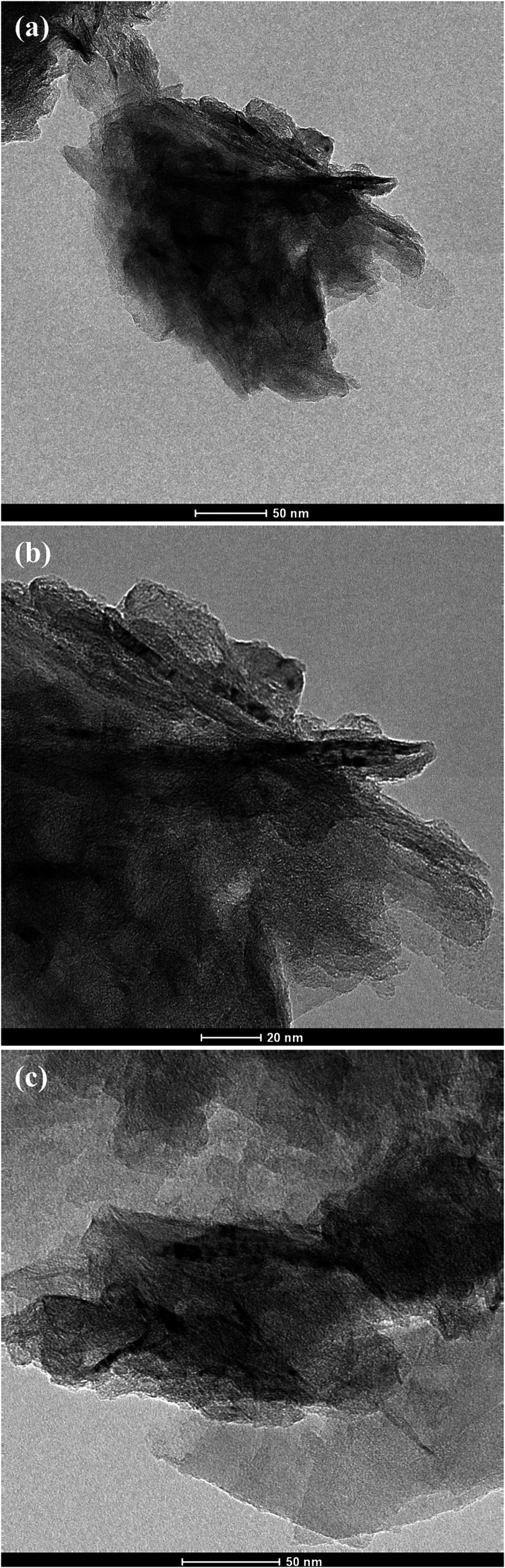
TEM analysis of graphene at magnifications of (a) 130,000×, (b) 255,000×, and (c) 180,000×. Source—Prepared by the authors (2025).

The micrographs in Figure 4(a,b) exhibit thin graphene sheets with good contrast and well-defined edges, suggesting a lamellar structure with a few layers or even monolayers. This morphology indicates good quality and dispersion of the material, as described by Ajala et al.^20^ Figure 4(c) presents partially overlapped graphene sheets with translucent areas indicative of few layers.^31^ The overlapping suggests irregular stacking, which is common in graphene obtained by chemical exfoliation, as discussed in refs 32,33 .

The SEM images of the characterized graphene, shown in Figure 5, reveal micrographs that allow visual analysis of the stacking of graphene sheets, their morphology, and the shapes suggested by their arrangement, demonstrating the material’s behavior.

**Figure 5:**
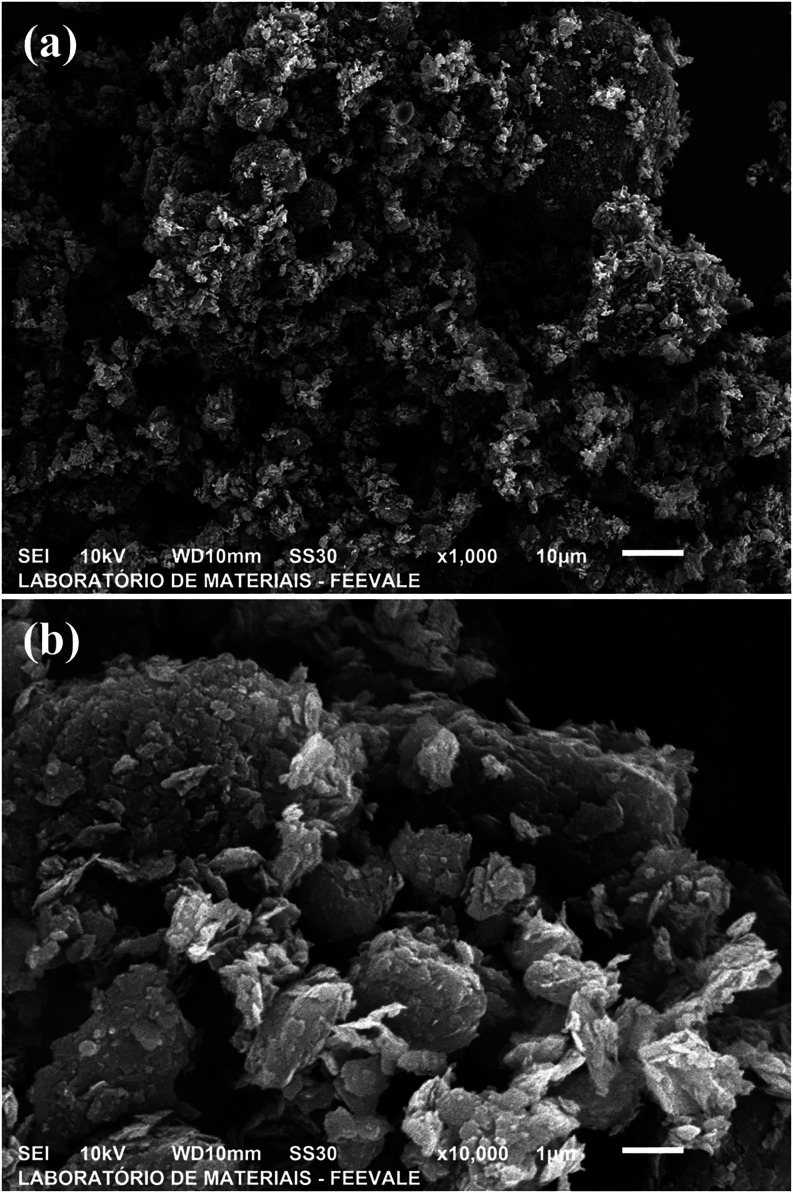
Scanning electron microscopy analyses of the graphene samples at magnifications of (a) 1000× and (b) 10,000×. Source—Prepared by the authors (2025).

The images reveal the characteristic graphene structures with surface roughness, agglomerates, and partially overlapped lamellar sheets. Irregular and folded structures are observed, which are consistent with few-layer graphene identified by Raman analysis, often prone to agglomeration due to its high surface area and the tendency of π–π interactions between sheets.^27^ This morphology may also result from structural defects, such as vacancies or residual oxygenated groups, which promote the formation of reactive edges and folds.^13^

In the case of graphene obtained by the mechanical exfoliation method, the irregular stacking observed in TEM and SEM images also directly influences the electronic and mechanical properties of the material. Although this method produces sheets of higher structural quality than chemical exfoliation, partially overlapping regions or misalignments between layers can still occur. From an electronic point of view, misalignments between sheets and turbostratic stacking reduce interlayer coupling, modifying electronic dispersion and decreasing the mobility of charge carriers.^34^

From a mechanical perspective, the presence of disordered overlapping layers hinders the behavior expected of a continuous monolayer, reducing the intrinsic stiffness and efficiency of stress transfer. A recent study^35^ has shown that variations in stacking order and interlayer sliding, such as those induced under uniaxial strain, significantly affect the mechanical response of few-layer graphene by altering interlayer coupling and load transfer efficiency under applied strain.

This morphology directly affects the final properties of the material, such as its dispersion capability in rubber matrices or performance in electrochemical applications. According to Zhang et al.,^36^ the presence of defects and the arrangement in thin layers enhance interfacial interaction with other materials, although they may partially compromise electrical conductivity. Therefore, the micrographs reinforce the structural interpretation suggested by Raman spectroscopy, contributing to an understanding of the 3D structure of the analyzed graphene.

### 3.2 Results of the Characterizations of the Developed Composites

The results of the FT-IR analysis are illustrated in Figure 6, allowing the visualization of the spectra obtained for each evaluated sample.

**Figure 6:**
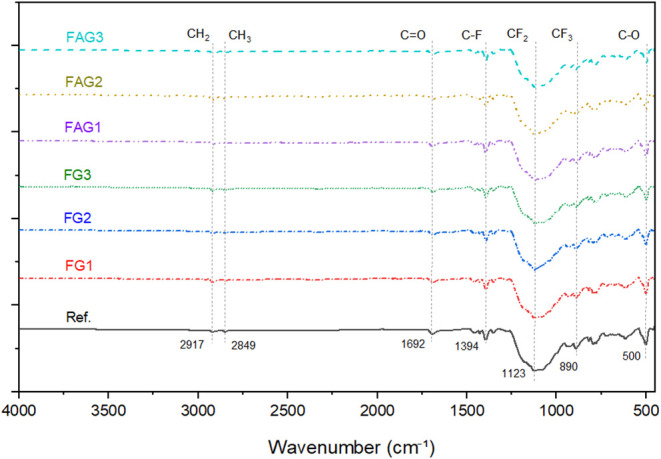
FT-IR spectra of the analyzed samples. Source—Prepared by the authors (2025).

The fluoroelastomer in an alkaline medium shows bands at 2917 and 2850 cm^–1^, attributed to CH_2_ and CH_3_ stretching, which are common in hydrocarbon chains present in copolymers or additives. The most characteristic bands of fluoroelastomers are those of C–F bonds, located between 1400 and 1000 cm^–1^, with particular emphasis on the asymmetric CF_2_ stretching, between 1150 and 1100 cm^–1^. Similar results were observed by Simon et al.^37^ in the analysis of thermo-oxidative aging of FKM rubber.

A comparison of the FT-IR spectra shows no absorption band around 2250 cm^–1^, associated with the nitrile group of acetonitrile in the graphene-containing composites. Although the FT-IR spectrum of pure acetonitrile was analyzed as a reference and clearly exhibited the characteristic C≡N stretching band in this region, such a band was not detected in the composite spectra. This confirms that acetonitrile was effectively removed during the drying process and did not chemically interact with the fluoroelastomer matrix or remain adsorbed on the graphene surface.

Thus, acetonitrile was employed with the primary purpose of improving the initial dispersion of graphene particles, which can enhance the intermolecular interactions between graphene and the fluoroelastomer matrix by increasing the effective contact area. However, this approach does not lead to chemical modification of graphene, and the interfacial interaction remains limited, since nonfunctionalized graphene exhibits low chemical affinity with fluorinated matrices. As reported by refs 14,15 , in the absence of functional groups capable of promoting stronger bonding, the graphene–FKM interface is mainly governed by weak van der Waals interactions, which restricts reinforcement efficiency.^38,39^

Figure 7 shows the scanning electron microscopy (SEM) images of the samples obtained at three different magnifications. These micrographs allow for the evaluation of the surface morphology, dispersion of phases, and possible structural differences between the reference material and the modified samples.

**Figure 7:**
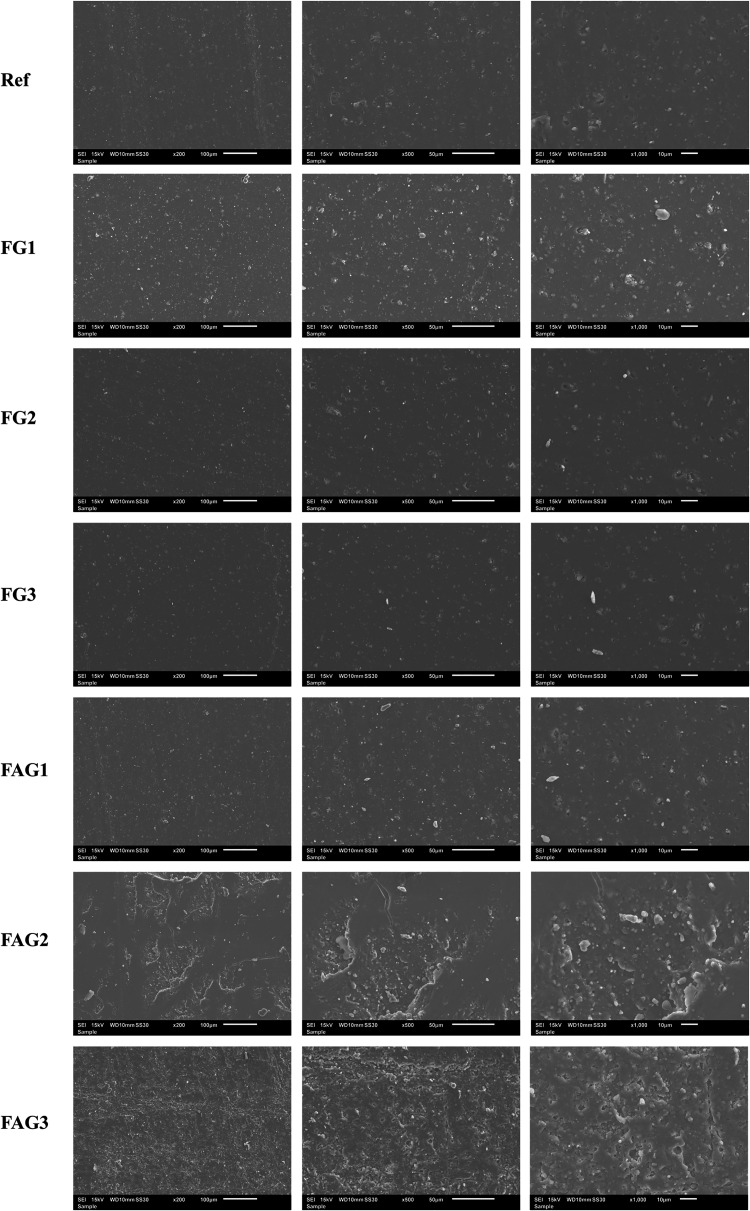
Scanning electron microscopy (SEM) analyses of the samples at three different magnifications. Source—Prepared by the authors (2025).

The SEM micrographs reveal differences in surface morphology among the analyzed samples. In particular, the samples processed with acetonitrile exhibit a more irregular and rougher surface compared to those prepared without a solvent. This increase in surface roughness suggests a more effective dispersion of graphene within the fluoroelastomer matrix, as a well-dispersed nanoscale filler tends to promote a more heterogeneous fracture surface.

The rough and textured morphology observed in the acetonitrile-assisted samples indicates improved interfacial interactions between graphene and the polymer matrix, reducing the formation of large agglomerates. Such morphological features are commonly associated with enhanced dispersion efficiency and are consistent with the role of solvent-assisted processing in facilitating a more uniform distribution of graphene throughout the material.

In a study by Liu et al.,^13^ SEM micrographs of graphene–elastomer composites exhibited surface morphologies comparable to those observed in the present work, characterized by relatively rough and heterogeneous fracture surfaces associated with a satisfactory dispersion of graphene within the matrix. Overall, the micrographs indicate that graphene is reasonably well dispersed in all of the analyzed samples, regardless of the processing route. More pronounced morphological differences, such as clear agglomeration or stronger textural contrast, would likely require the evaluation of higher graphene loadings, where dispersion limitations and interfacial effects would become more evident.

Figure 8 displays the EDS elemental mapping images of the analyzed samples, illustrating the spatial distribution of the detected elements and enabling a qualitative assessment of elemental homogeneity and the effectiveness of element incorporation within the matrix. In EDS mapping, each element must be interpreted according to the color assigned in its own result. The same element does not necessarily appear with the same color in different maps, since colors depend on the acquisition and visualization settings.

**Figure 8:**
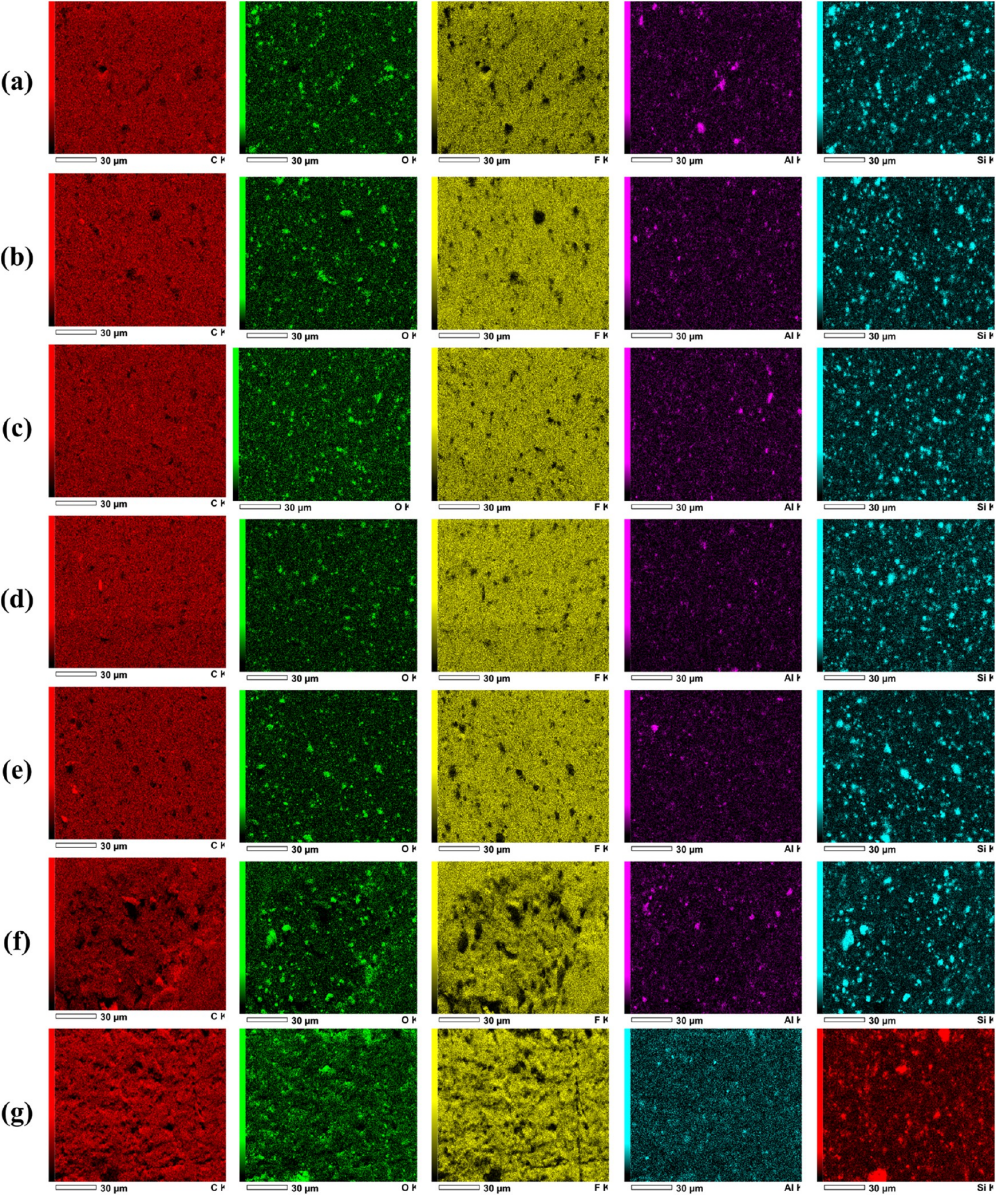
EDS elemental mapping images of the samples, showing the spatial distribution of the detected elements: (a) Reference, (b) FG1, (c) FG2, (d) FG3, (e) FAG1, (f) FAG2 et (g) FAG3. Source—Prepared by the authors (2025).

The EDS elemental mapping images were used as a qualitative tool to evaluate the spatial distribution and dispersion of carbonaceous phases within the analyzed surface. The maps reveal a continuous and homogeneous carbon signal with little evidence of localized regions of high intensity, indicating the absence of severe agglomeration in the samples containing graphene.

When comparing FG3 and FAG3, sample FAG3 exhibited a more uniform distribution of carbon across the analyzed area, even better than the other samples. This behavior suggests that the use of acetonitrile during processing promoted improved dispersion of graphene within the fluoroelastomer matrix, likely by facilitating better separation and distribution of the graphene sheets. These findings are consistent with the SEM observations and highlight the effectiveness of solvent-assisted processing in enhancing the dispersion of graphene.

Elemental mapping by EDS has been widely used as a complementary and qualitative technique to assess the spatial distribution and dispersion of elements in polymer-based composites. Recent studies have shown that EDS mapping is particularly effective in identifying elemental homogeneity and detecting localized enrichment or segregation, providing valuable insights into dispersion behavior even when the technique is not intended for quantitative analysis.^40–42^ In this context, EDS mapping serves as a supporting tool for morphological analyses, especially when combined with SEM observations, contributing to a comprehensive evaluation of the dispersion at the microscale.

The morphology of the composites with graphene incorporation depends on the chemical compatibility between the matrix and the filler. Xing et al.^22^ highlight that CF_3_ and CF_2_ groups are poorly reactive, hindering adhesion to nonfunctionalized graphene and favoring the formation of agglomerates. To improve dispersion, functionalization of graphene with vinyl or amine groups is an effective strategy. Xiong et al.^14^ showed that the use of functionalized graphene resulted in homogeneous morphology and good dispersion, reinforcing the importance of using different techniques to disperse graphene in the elastomeric matrix.

Dynamic mechanical analyses were conducted to investigate the viscoelastic properties, focusing on their behavior under different temperature conditions, as presented in Figure 9.

**Figure 9:**
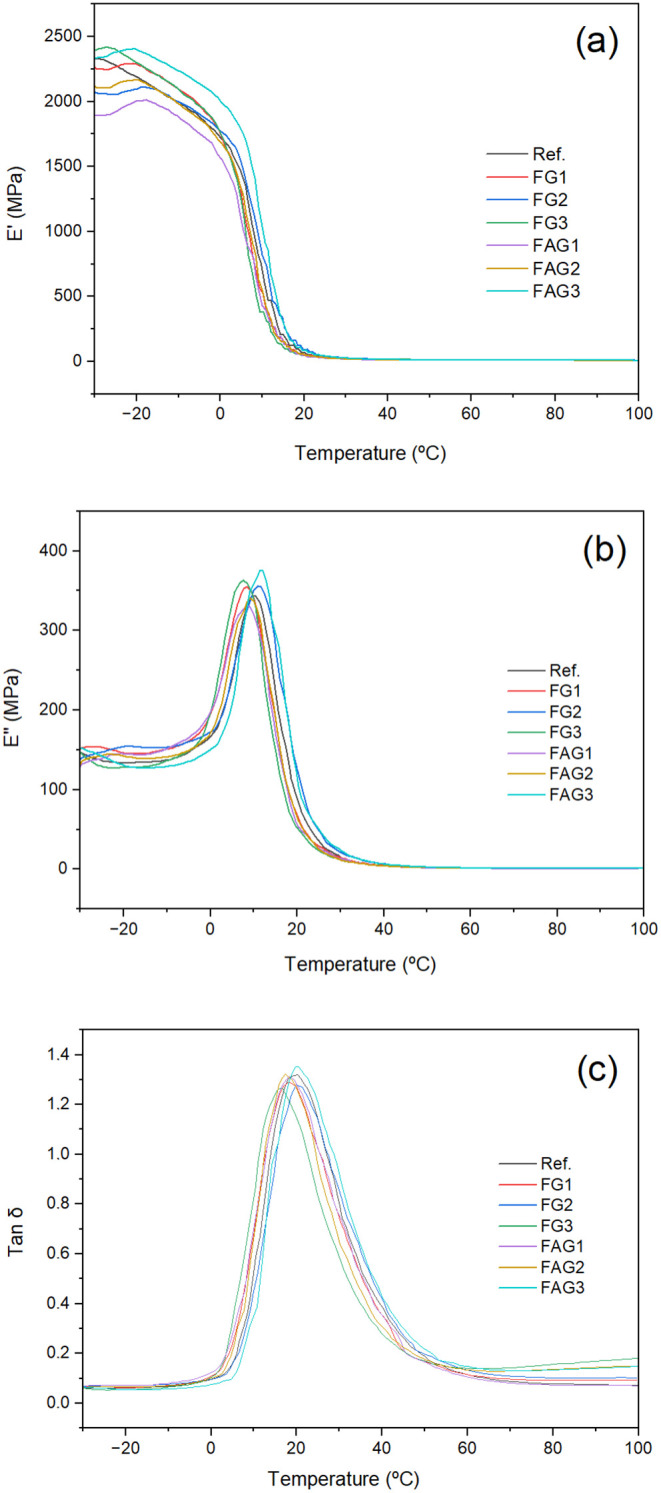
Dynamic mechanical analysis results showing the variation of (a) the storage modulus (*E*′), (b) the loss modulus (*E*″), and (c) the damping factor (tan δ). Source—Prepared by the authors (2025).

As the temperature approaches and exceeds the range of 0 °C to 20 °C, a significant reduction in the storage modulus (*E*′) is observed, indicating the transition from the glassy to the rubbery state. Within this range, the materials exhibit greater molecular mobility, establishing a plateau as the temperature increases.^22^

The loss modulus (*E*″) analyses show that all samples present a well-defined peak within the same temperature range associated with *T*_g_. This behavior reflects the maximum energy dissipation through molecular relaxation, followed by a sharp decrease at higher temperatures, characterizing the region dominated by the elastic behavior.

The intensity of the tan δ peak is related to the material’s ability to dissipate mechanical energy as heat.^43^ In this context, the FAG2 and FAG3 samples exhibited the highest tan δ values, with increases of 0.23% and 2.58%, respectively, compared with the reference sample.

Table 2 presents the results obtained from the TGA and DMA analyses of the studied samples. Thermal properties such as initial degradation temperature and glass transition behavior (*T*_g_) are described along with dynamic mechanical parameters.

**Table 2:** Results of the TGA and DMA Analyses^a^

sample	DTG_max_ (°C)	IDT (°C)	*T*_g_ (°C)	*E*′ at –30 °C (MPa)	tan δ (máx)
ref	551.18	340.34	10.00	2327	1.319
FG1	542.87	350.11	8.50	2259	1.291
FG2	551.18	448.61	11.20	2080	1.279
FG3	547.59	319.31	7.70	2392	1.264
FAG1	537.71	434.08	8.35	1892	1.313
FAG2	559.68	470.45	9.15	2110	1.322
FAG3	552.62	457.84	11.80	2333	1.353

a Source—Prepared by the authors (2025).

The results presented in Table 1 indicate significant differences in both the thermal behavior and the viscoelastic properties of the samples. It can be observed that the DTGmax ranges from 537.7 °C to 559.7 °C, with FAG2 exhibiting the highest thermal stability, while FAG1 shows the lowest value. The IDT also reflects this trend, with FAG2 and FG2 presenting higher values than the reference, indicating greater resistance to initial degradation.

^15^Wei et al.^15^ highlight that the presence of organic solvents, such as acetonitrile, can improve the compatibility between graphene and the matrix, promoting dispersion and interfacial interaction. This better integration contributes to the increased thermal stability of the composites, as graphene acts as a barrier that delays thermal degradation, hindering the release of volatile products.

Regarding *T*_g_, the values range from 7.7 °C to 11.8 °C, showing minor variations between the formulations; FAG3 presents the highest *T*_g_, suggesting a restriction effect on molecular mobility. The storage modulus (*E*′) at −30 °C indicates that FG3 and FAG3 maintain stiffness comparable to or higher than the reference, while FAG1 presents the lowest value, reflecting a reduction in matrix rigidity.^42^

The increase in *T*_g_ for the FAG3 sample suggests a restriction of segmental mobility associated with the interaction between the FKM chains and graphene nanoplatelets. Additionally, modifications in the tan δ response indicate a more heterogeneous glass transition, which can be related to the formation of interfacial regions with distinct chain dynamics within the composite.^43^

Concerning energy dissipation, represented by the tan δ peak, FAG3 exhibits the highest value, suggesting greater damping capacity, while FG3 and FG2 show lower values, indicating reduced dissipation. These results demonstrate that the addition of different fillers and treatments simultaneously influences the thermal stability, stiffness, and viscoelastic behavior of the samples.^14–17^

The use of acetonitrile as a dispersion method promotes a more homogeneous distribution of graphene throughout the FKM matrix, which intensifies the physical mechanisms of interfacial interaction responsible for the increase in the initial degradation temperature (IDT) observed in the FAG samples. Although pure graphene does not provide direct chemical compatibility with the fluoroelastomer, acetonitrile contributes to better interfacial contact by causing slight swelling of the fluorinated polymer and reducing surface tension, increasing the wettability of the graphene sheets.^44^

The improved wettability reduces agglomeration and expands the effective contact area of the filler, strengthening physical interactions, such as van der Waals forces and interfacial friction, between graphene and elastomeric chains. Similar solvent-assisted dispersion mechanisms have already been reported in FKM composites.^14,15^ The closer proximity between the phases restricts the mobility of the polymer chains in the region adjacent to the graphene, increasing the thermal stability of the system and delaying the onset of thermal decomposition, which could explain the higher IDT values obtained in the FAG samples.^45^

Figure 10 presents the correlation graph between the stress of the samples and their initial degradation temperature (IDT). This graph allows visualization of how mechanical resistance relates to thermal stability, highlighting trends among the different studied formulations.

**Figure 10:**
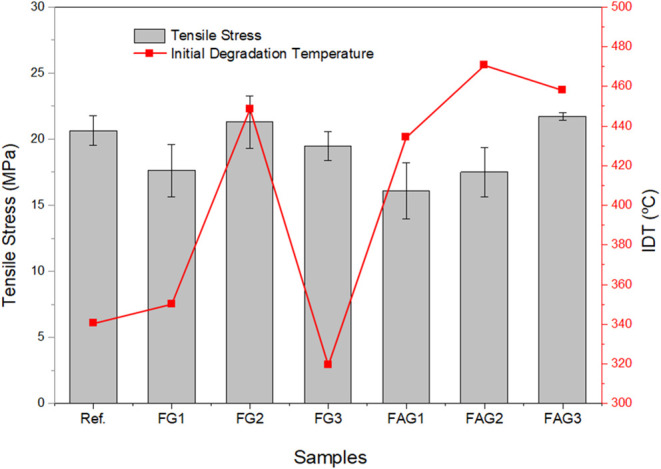
Correlation graph between stress and initial degradation temperature. Source—Prepared by the authors (2025).

It can be observed that, in general, samples with higher IDT, such as FG2, FAG2, and FAG3, tend to exhibit relatively higher stress values compared to the studied samples, with an increase in stress for the FAG3 sample of approximately 5.3%, suggesting that improvements in thermal stability may be associated with greater mechanical resistance.^46^ In contrast, FG3 presents the lowest IDT, accompanied by intermediate stress, indicating that modifications in the matrix can affect the thermal and mechanical properties. This behavior is consistent with studies reporting that the addition of fillers or matrix modifications can simultaneously optimize mechanical strength and thermal stability of polymers.^22^

It is observed that, although Liu et al.^13^ showed a more significant percentage increase in tensile strength with the incorporation of graphene, the direct comparison of absolute stress values must consider the formulation differences. In a study,^13^ composites containing only graphene, without carbon black, reached maximum stresses around 17 MPa, while the addition of 30 phr of carbon black increased the tensile strength to approximately 23 MPa, highlighting the strong reinforcing effect of this conventional filler. In the present study, the formulation already includes carbon black, resulting in higher stress values from the reference sample and reaching a maximum of 21.74 MPa for the FAG3 sample. Thus, although the relative increase promoted by graphene is smaller when compared to ref 13, the results indicate that the combination of carbon black and graphene, coupled with an efficient dispersion strategy, allows achieving tensile strength levels comparable to those reported in the literature, reinforcing the synergistic role between fillers in improving the mechanical properties of the fluoroelastomer.

Table 3 presents the results of the mechanical tests performed on the fluoroelastomer samples including Shore A hardness, stress, and elongation values.

**Table 3:** Results of the Mechanical Tests^a^

sample	shore A hardness	SD	tensile stress	SD	elongation (%)	SD
ref	80.55	2.40	20.65	1.12	258.00	11.63
FG1	72.00	0	17.63	1.98	364.00	10.97
FG2	77.33	2.87	21.30	1.98	295.00	18.25
FG3	72.33	0.50	19.49	1.09	479.50	57.87
FAG1	74.44	0.53	16.1	2.11	245.00	17.82
FAG2	77.44	4.64	17.51	1.88	301.00	32.50
FAG3	78.66	2.24	21.74	0.29	301.00	24.02

a Source—Prepared by the authors (2025).

Samples with graphene incorporated by the conventional method exhibit increased elongation, particularly FG3, suggesting a greater deformation capacity, while some samples with graphene incorporated using acetonitrile maintain or increase tensile strength such as FAG3. Hardness tends to slightly decrease with graphene incorporation, reflecting a balance between stiffness and flexibility, indicating that the type and method of incorporation directly impact the relationship between strength and deformability of the material.^47^

The sample FAG3 exhibits the best performance due to the efficient interfacial interaction between FKM and graphene. The simultaneous increase in *T*_g_ (11.8 °C) and tan δ (1.353) indicates greater restriction of segmental mobility but with good energy dissipation capacity, behaving like composites with a more homogeneous distribution. Furthermore, its superior IDT (457.84 °C) and DTGmax (552.62 °C) values demonstrate greater thermal stability, suggesting a molecular structure reinforced by better wettability and anchoring of graphene, as discussed in refs 15–17 for FKM/rGO composites.

In mechanical tests, FAG3 also stands out, exhibiting the highest tensile strength of 21.74 MPa and elongation of 301%, a balance generally associated with efficient load transfer and interfacial adhesion provided by the good dispersion of particles in the matrix or even by the intermolecular interaction between the matrix and the particle. Similar results were obtained by Li and Liao,^48^ who used molecular dynamics simulations to design epoxy nanocomposites reinforced with graphene quantum dots (GQDs) functionalized with oxygenated groups. They showed that these functionalized GQDs promote greater interaction between the nanofiller and the polymer matrix, resulting in substantial improvements in mechanical properties, such as increases in stiffness and resistance to deformation, attributed to greater dispersion and reduction of the free volume in the material, which favors efficient load transfer between phases. They conclude and emphasize, however, the importance of the type and location of the functional group in optimizing the composite properties.

Efficient graphene dispersion increases the effective surface area available for interaction with the elastomeric matrix, allowing graphene sheets to act as physical anchor points capable of intensifying intermolecular forces such as van der Waals interactions and interfacial friction. When individually distributed, these sheets create a wider and more energetically favorable contact network, restricting segmental mobility and favoring stress transfer in the composite. Maximizing the graphene surface area achieved by mitigating agglomeration is very important for strengthening interfacial coupling and promoting thermal and mechanical reinforcement even in the absence of functional groups.^49,50^

The observed simultaneous enhancement in thermal and mechanical properties is consistent with recent studies on carbon-based reinforcements in fluoroelastomer; although the present work focuses on graphene, correlations with other carbon allotropes help elucidate the underlying reinforcement mechanisms, which are primarily guided by efficient dispersion and strong interfacial interactions. Under these conditions, nanofillers restrict polymer chain mobility and enable effective stress transfer, while poorly dispersed systems tend to form agglomerates that limit reinforcement efficiency. In ref 50, the authors demonstrate that well-distributed nanofillers form an interconnected reinforcing network, in which graphene sheets contribute large interfacial contact areas while one-dimensional fillers assist in bridging and load transfer, effectively restricting chain mobility and enhancing stress distribution. These findings reinforce that the key factor governing reinforcement efficiency is not merely filler content but the quality of dispersion and interfacial contact.

The comprehensive analysis and correlations with the studies discussed throughout this work demonstrate that graphene predispersion or functionalization strategies improve its compatibility with the rubber matrix, leading to more efficient load transfer and enhanced thermal stability of the composites. Among the evaluated approaches, solvent-based predispersion proved to be the most effective route for graphene incorporation, enabling a more homogeneous dispersion and resulting in improvements in mechanical and thermal performance. These findings support the applicability of the developed composites in different systems, which require materials with high thermal stability, mechanical strength, and long-term reliability under aggressive service environments.

## 4 Conclusions

This study developed and characterized fluoroelastomer/graphene composites aimed at applications under severe service conditions. Two incorporation methods were compared, demonstrating that the graphene addition route plays a critical role in controlling dispersion, microstructural homogeneity, and final performance. FT-IR analyses confirmed that the chemical structure of the fluoroelastomer matrix remained unchanged, while SEM observations revealed the presence of agglomerates, particularly in the reference sample, highlighting the challenges associated with graphene dispersion. In contrast, composites prepared using acetonitrile exhibited improved filler distribution, resulting in enhanced thermal resistance, with increases of up to 38% in decomposition temperature, while preserving adequate viscoelastic behavior with superior energy dissipation observed for specific formulations.

Mechanical testing further evidenced the strong influence of the incorporation method on hardness and tensile performance with the best balance between strength and elongation achieved in samples processed via solvent-assisted dispersion, including a maximum tensile stress of 21.74 MPa. These results are consistent with recent studies on graphene reinforcements in fluoroelastomer matrices, indicating that the reinforcement mechanisms are greatly impacted by efficient dispersion and strong interfacial interactions.

Overall, the findings demonstrate that graphene can effectively enhance the thermal and mechanical properties of fluoroelastomers when the dispersion is properly controlled, making solvent-assisted incorporation a promising strategy for high-performance polymer composite applications.
